# Rg3 regulates myocardial pyruvate metabolism via P300-mediated dihydrolipoamide dehydrogenase 2-hydroxyisobutyrylation in TAC-induced cardiac hypertrophy

**DOI:** 10.1038/s41419-022-05516-y

**Published:** 2022-12-26

**Authors:** Jingyu Ni, Hao Zhang, Xiaodan Wang, Zhihao Liu, Tong Nie, Lan Li, Jing Su, Yan Zhu, Chuanrui Ma, Yuting Huang, Jingyuan Mao, Xiumei Gao, Guanwei Fan

**Affiliations:** 1 National Clinical Research Center for Chinese Medicine Acupuncture and Moxibustion, First Teaching Hospital of Tianjin University of Traditional Chinese Medicine, 300381 Tianjin, China; 2 Tianjin Key Laboratory of Translational Research of TCM Prescription and Syndrome, First Teaching Hospital of Tianjin University of Traditional Chinese Medicine, 300381 Tianjin, China; 3 Medical Experiment Center, First Teaching Hospital of Tianjin University of Traditional Chinese Medicine, 300381 Tianjin, China; 4 Haihe Laboratory of Modern Chinese Medicine, Tianjin University of Traditional Chinese Medicine, 301617 Tianjin, China; 5 Key Laboratory of Prevention and Treatment of Cardiovascular and Cerebrovascular Diseases of Ministry of Education, First Affiliated Hospital of Gannan Medical University, Gannan Medical University, 341000 Ganzhou, China

**Keywords:** Heart failure, Pharmacodynamics

## Abstract

The failing heart is characterized by an increase in glucose uptake and glycolytic rates that is not accompanied by a concomitant increase in glucose oxidation. Lower coupling of glucose oxidation to glycolysis possibly owes to unchanged or reduced pyruvate oxidation in mitochondria. Therefore, increasing pyruvate oxidation may lead to new therapies for heart disease. Dihydrolipoamide dehydrogenase (DLD) is a component of the pyruvate dehydrogenase complex (PDH). DLD mutations or defects are closely associated with metabolic diseases. However, few studies explore the effects of DLD mutants or acylation status on PDH activity and pyruvate metabolism. P300 is protein 2-hydroxyisobutyryltransferases in cells, and P300-dependent lysine 2-hydroxyisobutyrylation of glycolytic enzymes affects glucose metabolism. However, there are no relevant reports on the effect of 2-hydroxyisobutyrylation on the energy metabolism of heart failure, and it is worth further in-depth study. In this study, we showed that 2-hydroxyisobutyrylation is an essential protein translational modification (PTM) that regulates the activity of pyruvate dehydrogenase complex (PDHc). In a mouse model of transverse aortic constriction (TAC)-induced cardiac hypertrophy, the 2-hydroxyisobutylation of DLD was significantly increased, related to the decrease in PDH activity. In addition, our data provide clear evidence that DLD is a direct substrate of P300. As one of the main active ingredients of ginseng, ginsenoside Rg3 (Rg3) can reduce the 2-hydroxyisobutylation levels of DLD and restore the PDH activity by inhibiting the acyltransferase activity of P300, thereby producing beneficial effects whenever the heart is injured. Therefore, this study suggests a novel strategy for reversing myocardial hypertrophy.

## Introduction

Heart failure (HF) is characterized by a spectrum of alterations in cardiac energy and substrate metabolism [1, 2]. Metabolic remodeling is commonly observed in the failing myocardium, which extends from altered mitochondrial dynamics and function to altered substrate utilization and metabolism [3]. Glucose metabolism plays a potential role in ATP production and maintains diverse biochemical processes in cardiomyocytes. The failing heart is characterized by an increase in glucose uptake and glycolytic rates that is not accompanied by a concomitant increase in glucose oxidation. Indeed, experimental evidence strongly supports that lower coupling of glucose oxidation to glycolysis possibly owes to unchanged or reduced pyruvate oxidation in mitochondria [4–7]. Therefore, increasing pyruvate oxidation may lead to new therapies for heart disease.

Mitochondrial pyruvate metabolism is regulated by pyruvate dehydrogenase (PDH) [8]. PDH is a gatekeeper in pyruvate metabolism to maintain glucose homeostasis [9]. During HF, in addition to being regulated by pyruvate dehydrogenase phosphatase, pyruvate dehydrogenase kinase, and mitochondrial pyruvate carrier, PDH activity can also be regulated through protein post-translational modification [10–12]. Experimental evidence strongly supports that protein post-translational modifications plays an important role in regulating the activity and function of pyruvate dehydrogenase [10, 13–16]. Dihydrolipoamide dehydrogenase (DLD) is a component of the pyruvate dehydrogenase complex [17]. DLD mutations or defects are closely associated with metabolic diseases. Generally considered, DLD is a flavoenzyme that reversibly catalyzes the oxidation of reduced lipoyl substrates with the reduction of NAD^+^ to NADH. However, few studies explore the effects of DLD mutants or acylation status on PDH activity and pyruvate metabolism.

Protein lysine modifications play important roles in gene regulation, transcription, metabolism, and other biological processes [18]. Lysine 2-hydroxyisobutyrylation (Khib) in eukaryotes has recently been discovered as a novel protein modification, and this modification is associated with transcription, glycolysis, and other processes [19–22]. Research on histone lysine 2-hydroxyisobutyrylation was first published by Dai et al. in 2014 and gradually became a research hotspot in the following years [20]. Lysine 2-hydroxyisobutyrylation was first discovered in histone. However, with the development of proteomics, the in-depth identification of Khib substrate sites in mammalian cells by proteomics revealed that Khib modifications could occur on more than 6500 nonhistone proteins. At the same time, a systematic study of Khib’s “writer” and “Eraser” revealed that P300 and TIP60 were protein 2-hydroxyisobutyryltransferases, and HDAC2/3 were key protein de-2-hydroxyisobutyrylases in cells [19, 23, 24]. Increasing evidence showed that 2-hydroxyisobutyrylation played an essential role in metabolic processes. For example, it had been shown that Khib on K206 was a key regulatory modification of phosphoglycerate kinase enzymatic activity and mutation of the Khib site affected protein interactions of phosphoglycerate kinase and its substrates [25]. Huang et al. showed that P300 deficiency reduced the glycolysis and the cell survived [21]. However, there are no relevant reports on the effect of 2-hydroxyisobutyrylation on the energy metabolism of heart failure, and it is worth further in-depth study.

In this study, we showed that Rg3 protected mice from TAC-Induced cardiac hypertrophy and regulated pyruvate metabolism. We also found that 2-hydroxyisobutyrylation is an essential PTM that regulates the activity of PDH. In a mouse model of TAC-induced cardiac hypertrophy, the 2-hydroxyisobutylation of DLD was significantly increased, related to the decrease in PDH activity. In addition, our data provide clear evidence that DLD is a direct substrate of P300. Rg3 can reduce the 2-hydroxyisobutylation levels of DLD and restore the PDH activity by inhibiting the acyltransferase activity of P300, thereby producing beneficial effects whenever the heart is injured. Therefore, this study suggests a novel strategy for reversing myocardial hypertrophy.

## Results

### Rg3 protects mice from TAC-induced cardiac hypertrophy

**Fig. 1:**
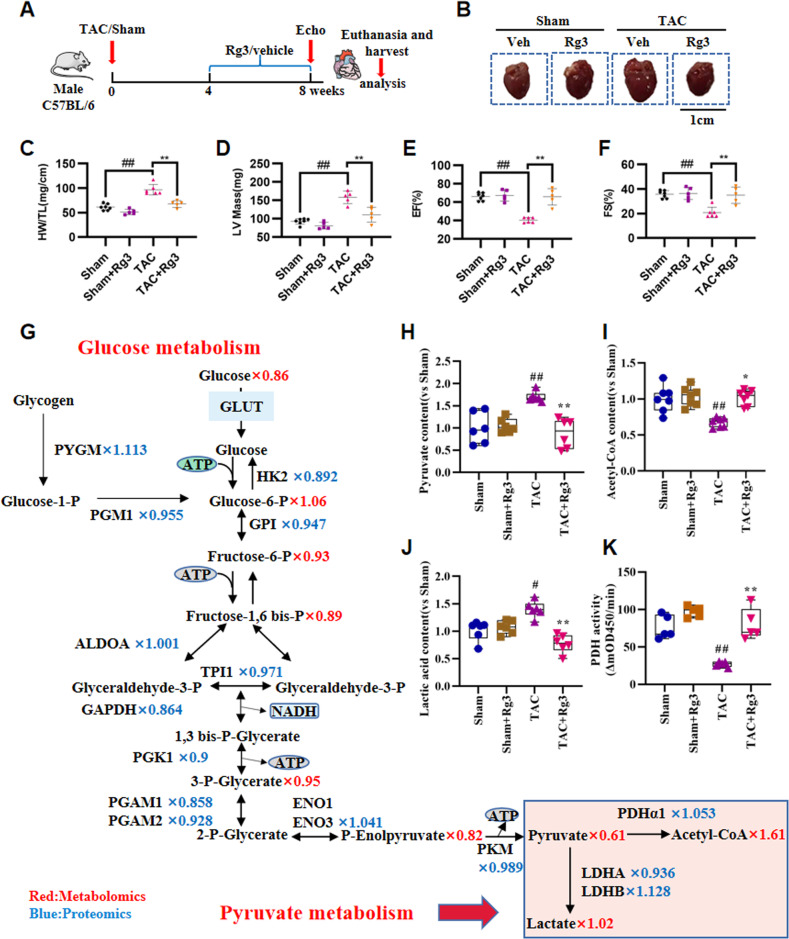
Rg3 Protects Mice from TAC-Induced Cardiac Hypertrophy and regulates pyruvate metabolism. **A** Schedule of animal treatments. Mice were subjected to TAC for 4 weeks, vehicle or Rg3 (20 mg/kg) was administered to TAC mice on the fourth week after surgery, and maintained by one administration per day for 4 weeks. **B** Image of the whole heart. **C** Heart weight to tibia length ratio, *n* = 5–8 for each group. **D**–**F** Quantification of left ventricular (LV) mass index (**D**), ejection fraction (EF%) (**E**), and fractional shortening (FS%) (**F**), *n* = 5–8 for each group. **G** Reconstruction of glucose metabolism from omics data. The identified proteins via proteomics and metabolomics were colored differently. The numbers in metabonomics and proteomics represented the change ratio of metabolites or enzymes of Rg3 treated compared with TAC group. **H**–**J** The contents of pyruvate, acetyl CoA, and lactate in heart tissue, *n* = 6 for each group. **K** The activity of pyruvate dehydrogenase in heart tissue, *n* = 5 for each group. All data were analyzed using one-way ANOVA and were expressed as means ± SD, ^#^*p* < 0.05, ^##^*p* < 0.01 versus Sham group, ^*^p < 0.05, ^**^p < 0.01 versus TAC group.

It is unknown whether Rg3 could directly modulate cardiac hypertrophy. To address this question, we examined the effects of Rg3 on a mouse hypertrophy model subjected to TAC for 4 weeks. Rg3 was administered to TAC mice the fourth week after surgery and maintained by one administration per day for 4 weeks (Fig. 1A). TAC induction resulted in significant cardiac hypertrophy compared to mice in the sham group, as estimated by heart weight to tibia length ratio (HW/TL). Rg3 treatment significantly reduced the size of the heart after TAC (Fig. 1B). Rg3 treatment significantly reversed the change in HW/TL by TAC (Fig. 1C). Moreover, multiple cardiac function-related echocardiographic parameters were significantly improved in the Rg3-treated group compared to the TAC group, including left ventricular (LV) mass index, ejection fraction (EF%), and fractional shortening (FS%) (Fig. 1D–F).

### Rg3 regulates pyruvate metabolism

Our prior work demonstrated that Rg3 regulated glucose metabolism [26]. Therefore, we reconstructed the myocardial glucose metabolic pathways and integrated the proteomics and metabolomics data to systematically overview the myocardial glucose metabolism process (Fig. 1G). It was found that pyruvate metabolism changed most significantly in the glucose metabolic pathway. The content of pyruvate, lactate, and acetyl-CoA in the heart tissue were measured to validate the impact of Rg3 on pyruvate metabolism. The results showed that pyruvate and lactate content increased significantly, and acetyl-CoA content significantly decreased in the TAC model compared with the Sham group. Rg3 treatment significantly decreased the pyruvate and lactate content and increased the acetyl-CoA content (Fig. 1H–J). Next, the activity of pyruvate dehydrogenase was measured. The results showed that PDH activity was significantly decreased in the TAC model compared to the Sham group. Rg3 treatment significantly increased PDH activity (Fig. 1K). Our results suggested that Rg3 regulated pyruvate metabolism in TAC-induced cardiac hypertrophy.

### Protein lysine 2-hydroxyisobutyrylation exerts changes in the Rg3-treated TAC-induced cardiac hypertrophy

**Fig. 2:**
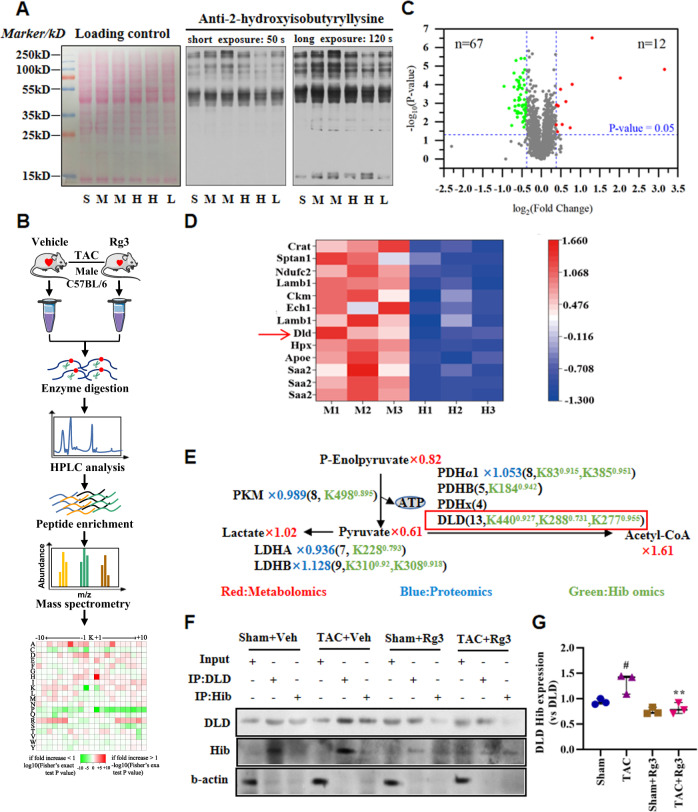
Protein Lysine 2-Hydroxyisobutyrylation Exerts Changes in the Rg3-treated TAC-Induced Cardiac Hypertrophy. **A** Western blot of different tissue lysates against anti-2-hydroxyisobutyryllysine antibody, *n* = 2–3 for each group. S: Sham, M: TAC, H: Rg3-20mg/kg group, L: Rg3-10mg/kg group. **B** The analytical strategy and method for global profiling of Khib on the TAC group and the Rg3-treated group by combining the affinity enrichment and proteomics techniques. **C** Volcano Chart Analysis of the Khib sites for the TAC group and the Rg3-treated group, *n* = 3 for each group. **D** The heat map showed the the 10 proteins with the most remarkable down-regulation by 2-hydroxyisobutyrylation. M: TAC, H: Rg3-20mg/kg group. **E** Reconstruction of glucose metabolism from omics data. The numbers in metabonomics and proteomics represented the change ratio of metabolites or enzymes of Rg3 treated compared with TAC group. **F**, **G** Heart homogenates were immunoprecipitated with anti-DLD or anti-2-hydroxyisobutyryl-lysine agarose beads, and probed with anti-2-hydroxyisobutyryl-lysine, anti-DLD, and anti-β-actin antibody. The representative immunoblots were presented, *n* = 3 for each group. The data were analyzed using Kruskal wallis test, ^#^*p* < 0.05, ^##^*p* < 0.01 versus Sham group, *p < 0.05, **p < 0.01 versus TAC group.

Lysine 2-hydroxyisobutyrylation is closely correlated with glucose metabolism [21, 27–29]. However, it is still unclear whether Rg3 regulates glucose metabolism through regulating lysine 2-hydroxyisobutyrylation. Towards this goal, we first performed a detection of lysine 2-hydroxyisobutyrylation in the heart. Ponceau staining was used to verify that the transfer efficiency and total amount of protein loaded were consistent. The results showed that the total amount of protein loaded in each well was the same. To examine the abundance and distribution of lysine 2-hydroxyisobutyrylation in the heart, we used anti-2-hydroxyisobutyryl lysine antibody to perform the western blot assay. The experimental results showed that the lysine 2-hydroxyisobutylation of the heart tissue of the TAC group changed when compared with the sham group. Administration of Rg3 affected the levels of lysine 2-hydroxyisobutyrylation in heart tissue compared with the TAC group (Fig. 2A).

We performed a systematic PTM analysis on the TAC group and the Rg3-treated group to identify potential modified proteins and sites by combining affinity enrichment and proteomics techniques. The research process and strategy were illustrated in Fig. 2B. In this study, 3157 2-hydroxyisobutylation sites on 581 proteins were identified, of which 2639 sites on 515 proteins contained quantitative information. The effect of protein expression abundance on modification levels was eliminated after proteomic normalization, 2575 sites of 471 proteins containing quantitative information were identified. Among the 471 proteins 2-hydroxyisobutyrylated, there were 79 sites and 27 proteins identified that were over- or under-expressed significantly with expression changes more than 1.3-fold at *P* < 0.05 in the comparison between the TAC group and the Rg3-treated group. 12 sites and 8 proteins were upregulated, 67 sites and 19 proteins were downregulated in response to Rg3 treatment (Fig. 2C).

We investigated the effect of Rg3 on the lysine 2-hydroxyisobutyrylation sites of TAC-induced cardiac hypertrophy mice, which may be related to the regulation of Rg3 in pyruvate metabolism. The 10 proteins with the most remarkable down-regulation by 2-hydroxyisobutyrylation are shown as a heat map. Among the downregulated proteins, the proteins located in the mitochondria were DLD, Ech1, and Crat (ordered by changes from high to low) (Fig. 2D).

Then, we reconstructed the myocardial glucose metabolic pathways and integrated the proteomics, 2-hydroxyisobutyrylation proteomics, and metabolomics data to systematically overview the myocardial glucose metabolism process (Fig. 2E). It was found that DLD protein, an essential component of the pyruvate dehydrogenase complex, had the most significant difference among the differentially expressed proteins. Therefore, DLD was identified as a potential target. We next sought to verify that Rg3 reduced the 2-hydroxyisobutylation level of DLD in TAC-induced cardiac hypertrophy. 2-Hydroxyisobutyrylation of DLD was significantly enhanced after TAC, and the elevated 2-hydroxyisobutyrylation level of DLD was significantly reduced by Rg3 administration (Fig. 2F, G).

### The change of 2-hydroxyisobutylation level at K440 is a key point in the regulation of pyruvate metabolism by Rg3

**Fig. 3:**
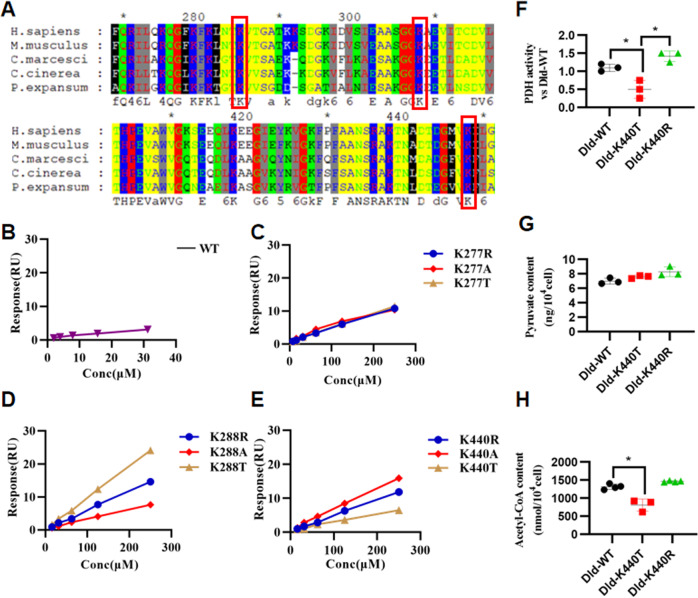
The change of 2-hydroxyisobutylation level at K440 is a key point in the regulation of pyruvate metabolism by Rg3. **A** Multiple sequence alignment of DLD from different species. The conserved K277, K288, and K440 site were marked by the red boxes. **B**–**E** The binding ability of Rg3 with the DLD protein with or without site-directed mutagenesis were determined by surface plasmon resonance analysis. **F** The activity of pyruvate dehydrogenase in HEK293 cell with or without site-directed mutagenesis of DLD. **G**, **H** The contents of pyruvate and acetyl-CoA in HEK293 cell with or without site-directed mutagenesis of DLD. The data were analyzed using Kruskal Wallis test and were expressed as means ± SD, *p < 0.05.

Based on sequence alignment, K277, K288, and K440 of DLD were conserved in different species, indicating that K277, K288, and K440 might be important for an evolutionarily conserved function (Fig. 3A). Moreover, we locked 2-hydroxyisobutyrylated K277, K288, and K440 of DLD by threonine (replacing K by T), non-2-hydroxyisobutyrylated K277, K288, and K440 of DLD by arginine (replacing K by R), and unmodified states K277, K288, and K440 of DLD by alanine (replacing K by A). We cloned, overexpressed, and purified DLD (DLD-K277T, DLD-K277A, DLD-K277R, DLD-K288T, DLD-K288A, DLD-K288R, DLD-K440T, DLD-K440A, and DLD-K440R) as His-tagged fusion proteins. Surface plasmon resonance technology was used to explore the effect of changes in the level of 2-hydroxyisobutyrylation on the binding ability between Rg3 and DLD. The results showed that the level of 2-hydroxyisobutyrylation at K288 and K440 affected the binding ability between Rg3 and DLD (Fig. 3B–E). Among them, K440 is located in the dimerization domain of the pyridine nucleotide-disulfide oxidoreductase of the DLD protein, which can interact with PDHx and is important for the activity of the pyruvate dehydrogenase complex. To further investigate the effect of 2-hydroxyisobutyrylation modification status at K440 of DLD protein on the pyruvate metabolic pathway, we overexpressed Dld-WT, K440R, and K440T in HEK293 cells. The results showed that the 2-hydroxyisobutyrylation status of the K440 site of DLD protein affected the activity of PDH. Taken together, the results indicated that the change of 2-hydroxyisobutylation level at K440 was a key point in the regulation of pyruvate metabolism by Rg3 (Fig. 3F–H).

### P300 mediates DLD 2-hydroxyisobutyrylation

**Fig. 4:**
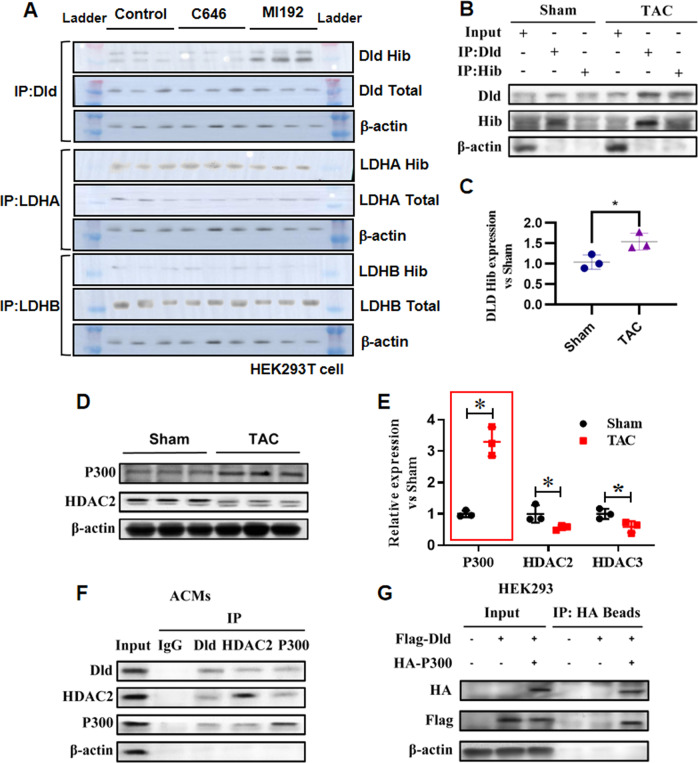
P300 mediates DLD 2-hydroxyisobutyrylation. **A** HEK293 cells, with or without C646, were transfected with indicated plasmid and harvested for immunoprecipitation and immunoblot analysis with the indicated antibodies. The representative immnoublots were presented, *n* = 3 for each group. **B**, **C** Heart homogenates of the Sham and TAC group were immunoprecipitated with anti-DLD or anti-2-hydroxyisobutyryl-lysine agarose beads, and probed with anti-2-hydroxyisobutyryl-lysine, anti-DLD, and anti-β-actin antibody. The representative immnoublots were presented, *n* = 3 for each group. **D**, **E** Representative digital western blots of P300, HDAC2, and HDAC3, and corresponding quantitative data, *n* = 3 for each group. **F** The reciprocal immunoprecipitaiton experiment showed that DLD and P300 were complexed in ACMs, *n* = 3 for each group. **G** DLD and P300 constituted a complex in the HEK293 cells, *n* = 3 for each group. The data were analyzed using Kruskal Wallis test and were expressed as means ± SD, *p < 0.05.

Studies have found that P300 was a “writer” for lysine 2-hydroxyisobutyrylation and P300-mediated lysine 2-hydroxyisobutyrylation regulated glycolysis in HCT116 cells [21]. It is unknown whether P300 directly modulates DLD lysine 2-hydroxyisobutyrylation. To address this question, we used C646 to inhibit the HAT activity of p300 in HEK293T cells. The results showed that inhibition of P300 HAT activity reduced the level of 2-hydroxyisobutyrylation of DLD. In addition, lactate is also an important metabolite of pyruvate metabolism, in which LDHA and LDHB play an important role. Therefore, we also investigated whether inhibition of P300 HAT activity affects this process. The results showed that inhibition of P300 HAT activity had no significant effect on the total protein levels and 2-hydroxyisobutyrylation levels of LDHA and LDHB (Fig. 4A). Heart samples obtained from the left ventricle of mice in the TAC group and sham group were analyzed by Co-Immunoprecipitation. The 2-hydroxyisobutyrylated DLD was markedly higher in the TAC-induced hypertrophic myocardium than in the sham group (Fig. 4B, C).

P300 protein level was detected by immunoblotting. The results showed that the P300 protein level increased significantly after TAC (Fig. 4D, E). The results preliminarily suggested that P300 might regulate the level of 2-hydroxyisobutyrylation of DLD. Next, we verified whether the regulation of the level of 2-hydroxyisobutyrylation of DLD was due to the interaction between P300 and DLD. To verify this interaction, lysates of mouse adult cardiomyocytes (ACMs) were immunoprecipitated with an anti-DLD antibody and probed with an anti-P300 antibody. Inversely, the ACM lysates were immunoprecipitated with an anti-P300 antibody and probed with an anti-DLD antibody. This reciprocal immunoprecipitation experiment showed that DLD and P300 were complex in ACMs (Fig. 4F). HEK293 cells were transfected with expression plasmids for HA-P300 and Flag-DLD, and the cell lysates were subjected to reciprocal immunoprecipitation experiments. The results indicated that DLD and P300 were complex in HEK293 cells (Fig. 4G). The data showed that P300 directly interacted with DLD and raised the possibility that P300 regulated the 2-hydroxyisobutyrylation of DLD.

**Fig. 5:**
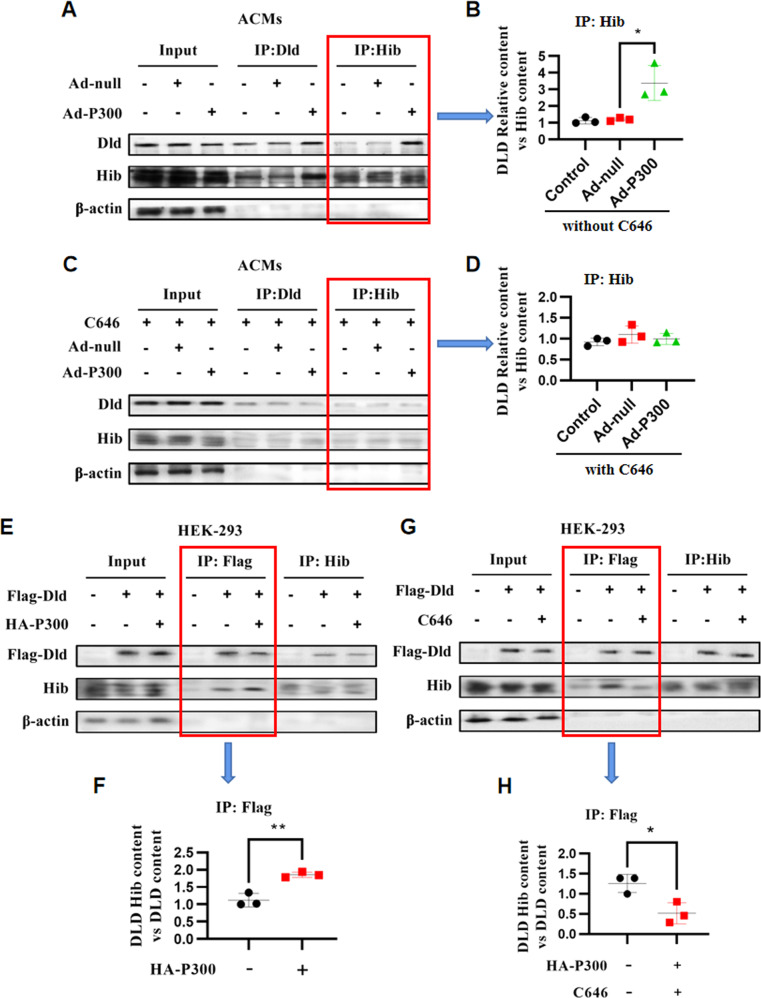
P300 mediates DLD 2-hydroxyisobutyrylation. **A**, **B** ACMs cells were transfected with or without P300 plasmid and harvested for immunoprecipitation and immunoblot analysis with the indicated antibodies, *n* = 3 for each group. **C**, **D** After treatment with P300 inhibitor C646, ACMs cells were transfected with or without P300 plasmid and harvested for immunoprecipitation and immunoblot analysis with the indicated antibodies, *n* = 3 for each group. **E**, **F** HEK293 cells were transfected with or without P300 plasmid and harvested for immunoprecipitation and immunoblot analysis with the indicated antibodies, *n* = 3 for each group. **G**, **H** After treatment with C646, HEK293 cells were transfected with or without P300 plasmid and harvested for immunoprecipitation and immunoblot analysis with the indicated antibodies, *n* = 3 for each group. The data were analyzed using Kruskal–Wallis test and were expressed as means ± SD, *p < 0.05.

We next sought to determine the role of P300 in 2-hydroxyisobutyrylation of DLD. In ACMs, 2-hydroxyisobutyrylation of DLD was significantly increased by plasmid-mediated overexpression of P300 (Fig. 5A, B), and the increased 2-hydroxyisobutyrylation of DLD was significantly reduced when treated simultaneously with C646 (P300-HAT inhibitor) (Fig. 5C, D). Flag-DLD expressed in HEK293 cells was 2-hydroxyisobutyrylation, as determined by immunoprecipitation with an anti-Flag antibody followed by probing with a 2-hydroxyisobutyrylation antibody. 2-Hydroxyisobutyrylation of Flag-DLD was significantly increased when HA-P300 was co-expressed (Fig. 5E, F), whereas it was significantly reduced in cells where P300 expression was inhibited by C646 (Fig. 5G, H). Reciprocal immunoprecipitation experiments consistently showed that P300 regulated DLD 2-hydroxyisobutyrylation.

### Rg3 Downregulates DLD 2-Hydroxyisobutyrylation and Regulates Pyruvate Metabolism by P300

**Fig. 6:**
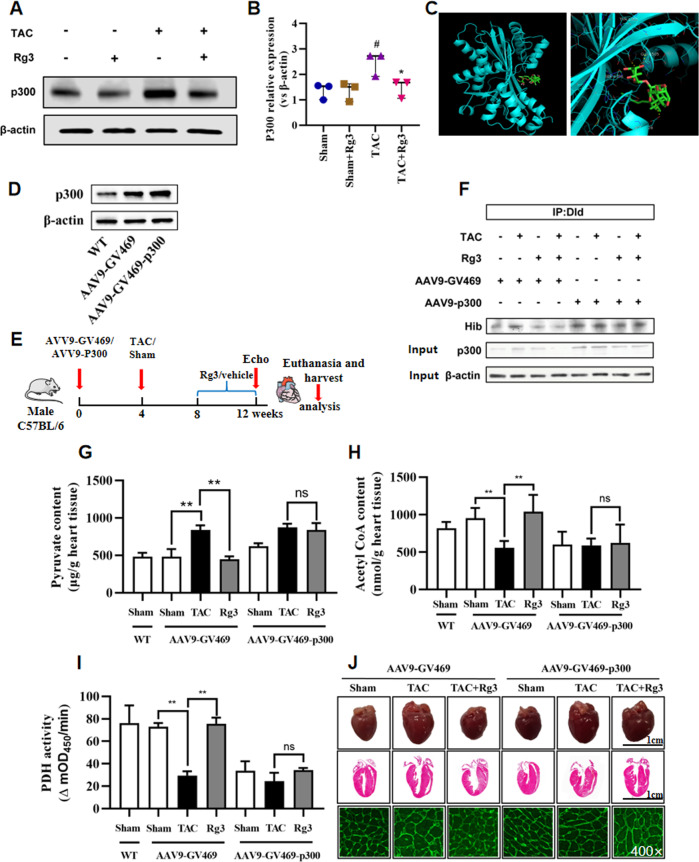
Rg3 Downregulated DLD 2-Hydroxyisobutyrylation and Regulated Pyruvate Metabolism by P300. **A**, **B** Representative digital western blots for P300 and β-actin, and corresponding quantitative data, *n* = 3 for each group. **C** Molecular docking showing Rg3 in the binding site of P300. **D** Representative digital western blots for P300 and β-actin, *n* = 3 for each group. **E** Schedule of animal treatments. AVV9-GV469 and AVV9-GV469-P300 were injected intracardiacally. TAC operation was performed four weeks later. Rg3 or vehicle was administered to TAC mice the fourth week after surgery and maintained by one administration per day for 4 weeks. **F** Heart homogenates were immunoprecipitated with anti-DLD agarose beads, and probed with anti-2-hydroxyisobutyryl-lysine, anti-DLD, and anti-β-actin antibody. The representative immnoublots were presented, *n* = 3 for each group. **G**, **H** The contents of pyruvate and acetyl-CoA in heart tissue, *n* = 3 for each group. **I** The activity of pyruvate dehydrogenase in heart tissue, *n* = 3 for each group. **J** Representative images showing gross cardiac morphology of mice in each group. HE staining of longitudinal heart. WGA staining, *n* = 5–8 for each group. The data were analyzed using Kruskal–Wallis test and were expressed as means ± SD, ^#^*p* < 0.05, ^##^*p* < 0.01 versus Sham group, *p < 0.05, **p < 0.01 versus TAC group.

Based on previous experiments, we investigated whether Rg3 regulated DLD 2-hydroxyisobutyrylation and regulated pyruvate metabolism via p300. Rg3 reduced the protein level of P300 after TAC (Fig. 6A, B). Molecular docking showed that Rg3 bound well to P300 (Fig. 6C). Then, we generated AAV9 viruses encoding a flag-tagged open reading frame of mouse P300, thus inducing P300 overexpression (Fig. 6D). AVV9-GV469 and AVV9-GV469-P300 were injected intracardially. TAC operation was performed four weeks later. Rg3 or vehicle was administered to TAC mice the fourth week after surgery and maintained by one administration per day for 4 weeks (Fig. 6E). DLD 2-hydroxyisobutyrylation was not reduced by Rg3 when P300 is overexpressed (Fig. 6F). In these mice, the increased pyruvate content and the decreased acetyl-CoA content or PDH activity were not restored by Rg3 when P300 was overexpressed (Fig. 6G–I). Myocardial hypertrophy was not rescued by Rg3 when P300 was overexpressed (Fig. 6J).

The data demonstrated that Rg3 downregulated DLD 2-hydroxyisobutyrylation and regulated pyruvate metabolism by P300.

## Discussion

Lysine acylation is a reversible post-translational modification of proteins that affects the enzyme activity, DNA binding capacity, and protein stability by changing the charge on lysine residues and protein structure. At the same time, lysine acylation can also regulate the activity of many nonhistone proteins, thereby regulating cell survival, gene transcription, signal transduction, and metabolism of mammalian cells [30, 31]. In recent years, it has been discovered that cardiovascular disease or cardiovascular disease risk factors are significantly correlated with the level of lysine acylation modification [30]. For example, studies have shown that high levels of histone lysine propionylation, butylation, and crotonylation are associated with obesity [32–34], high levels of lysine propionylation are associated with diabetes [35], and high levels of lysine succinylation are associated with myocardial ischemia-reperfusion injury [35, 36]. However, there is no relevant research on the role of new lysine acylation in myocardial hypertrophy and the role of small molecule drugs. In this study, we focused on 2-hydroxyisobutyrylation, which is closely related to the regulation of glucose metabolism. It was found that protein lysine 2-hydroxyisobutyrylation exerted changes in the Rg3-treated TAC-induced cardiac hypertrophy. This study suggested a novel strategy for reversing myocardial hypertrophy.

Through 2-hydroxyisobutyryl quantitative omics, three differentially changed lysine sites in DLD protein regulated by ginsenoside Rg3 were screened out. The K440 site is located in the dimerization domain of the pyridine nucleotide-disulfide oxidoreductase of the DLD protein. Multiple sites in this domain can interact with PDHX and are important for the activity of the pyruvate dehydrogenase complex. The level of 2-hydroxyisobutyrylation at K440 can affect the binding ability of ginsenoside Rg3 and DLD protein. To further investigate the effect of 2-hydroxyisobutyrylation modification status at K440 of DLD protein on the pyruvate metabolic pathway, we overexpressed Dld-WT, K440R, and K440T in HEK293 cells. The results showed that the 2-hydroxyisobutyrylation status of the K440 site of DLD protein affects the activity of PDH. Based on this, we speculate that the role of ginsenoside Rg3 in regulating pyruvate metabolism depends on the level of 2-hydroxyisobutyrylation modification at this site. Therefore, in this study, we used activity of pyruvate dehydrogenase and its related pyruvate and acetyl-CoA contents as the detected phenotype. However, we found an interesting phenomenon that pyruvate content remained unchanged under three conditions (Fig. 3G), while acetyl coenzyme A content decreased under Dld-K440T. This was because the cell culture medium contained pyruvate, so there was no significant difference in the influence on the pyruvate content. Moreover, it was found that DLD activity is not generally tested to evaluate its function; therefore, DLD activity was not tested.

DLD protein is not only an important component of the pyruvate dehydrogenase complex (PDHc), but is also a common component in α-ketoglutarate (KGDHc) and branched chain (BCKDH) α-keto acid dehydrogenase complexes. It is also part of the glycine cleavage system [37]. In this study, combined with the experimental results of other omics, we only investigated the role of DLD protein in the pyruvate dehydrogenase complex. However, the role of DLD in several other compounds is also worthy of further study, which is also a limitation of this research.

This study also explored the potential mechanism of regulating the 2-hydroxyisobutyrylation of DLD protein. Lysine 2-hydroxyisobutyrylation is a reversible protein post-translational modification regulated by lysine acyltransferases (KATs) and lysine deacetylating enzymes (KDACs). Enzymes that can regulate Khib modifications have also been identified. P300, Tip60, and CobB can be used as Khib modification enzymes; HDAC2 and HDAC3 can be used as Khib demodification enzymes. Previous studies have shown that deletion of p300 significantly reduces Khib levels on several p300-dependent, Khib-specific sites on key glycolytic enzymes, including ENO1, decreasing their catalytic activities [21, 29]. Previous studies have shown that the overexpression of P300 stimulates cardiomyocyte hypertrophy, and inhibition of P300 activity can prevent cardiomyocyte hypertrophy, which clearly indicates that P300 activity plays a direct role in regulating pathological myocardial hypertrophy [38, 39]. In this study, we also found that the level of P300 protein increased after TAC and decreased after Rg3 treatment. Therefore, we investigated the effects of P300 on the level of 2-hydroxyisobutyrylation of DLD protein. The results confirmed that P300-mediated DLD 2-hydroxyisobutyrylation. Based on the above results, we further explored whether ginsenoside Rg3 modulates the level of 2-hydroxyisobutyrylation of DLD protein dependent on P300. The results showed that Rg3 downregulated DLD 2-hydroxyisobutyrylation and regulated pyruvate metabolism by P300. However, P300 overexpression alone did not affect the phenotype related to mouse cardiac function, which may be due to the short time of P300 overexpression and the lack of phenotypic changes. Later, the time of P300 overexpression should be extended to further investigate the effect of P300 overexpression on mouse cardiac function related phenotypes. Here, because the molecular weight of P300 was too large and the gene sequence was too long to construct a stable P300 knockdown adeno-associated virus, only P300 overexpression adeno-associated virus was constructed. In subsequent studies, other methods should be used to knock down P300 to confirm further the role of P300 in ginsenoside Rg3 in regulating the 2-hydroxyisobutylation level of DLD protein and reducing TAC-induced cardiac hypertrophy in mice.

In conclusion, protein lysine 2-hydroxyisobutyrylation exerted changes in the Rg3-treated TAC-induced cardiac hypertrophy. The glucose metabolism pathway was comprehensively described by integrating the data of myocardial tissue proteomics, myocardial tissue 2-hydroxyisobutyryl quantitative omics, plasma metabolomics, and myocardial tissue targeted metabolomics. It has been demonstrated that TAC-induced up-regulation of DLD protein 2-hydroxyisobutyrylation is related to the up-regulation of p300 expression in the heart. Ginsenoside Rg3 downregulated DLD 2-hydroxyisobutyrylation and regulated pyruvate metabolism by P300. However, the specific effect of P300 on DLD and the specific mechanism regulating glucose and fatty acid metabolism is still unclear. The mechanism and regulatory sites of 2-hydroxyisobutyrylation still need to be studied further. However, these research results still provide a new perspective for a better understanding and explaining the mechanism of medicine in the treatment of cardiovascular diseases, including myocardial hypertrophy, and provide a new research perspective and theoretical basis for the targeted therapy of cardiovascular diseases.

## Materials and methods

### TAC model preparation and drug administration

Studies were conducted using the male C57BL/6 J mice aged 8–10 weeks (weight, 18–22 g) purchased from the Beijing Vital River Laboratory Animal Technology Co., Ltd. (Beijing, China). One hundred and ten mice were housed individually under standard conditions with a 12-h light/dark cycle and received a standard diet and water. The experimental procedures conformed to the Directive 2010/63/EU of the European Parliament, and all animals were handled according to the guidelines of the TCM Animal Research Committee (TCM-LAEC2014005) of Tianjin University of Traditional Chinese Medicine. The mice were anesthetized with an intraperitoneal injection of 15 mg/mL tribromoethanol (30 mg/kg, Sigma, Darmstadt, Germany), and the model of heart failure was induced via TAC, as described previously [40, 41]. Four weeks after TAC surgery, the mice were divided into group. When grouping, the heart EF of mice must be controlled between 38–50%, and they were randomly divided into TAC group and TAC + Rg3 group. Ginsenoside Rg3 (Shanghai Winherb Medical Technology Co., Ltd., Shanghai, China) was suspended in normal saline intraperitoneally (20 mg/kg/day or 10 mg/kg/day). Mice in the sham and TAC groups were administered saline at an equal volume via intraperitoneal injections. Twenty mice in each group were treated once a day. The dosage of Rg3-10mg/kg (L) is only involved in Fig. 2A, and the dosage of other Rg3 is 20 mg/kg.

### Quantitative proteome analysis based on LC-MS/MS

Protein extraction, trypsin digestion, TMT labeling, HPLC fractionation, Khib peptides affinity enrichment, and LC-MS/MS analysis according to previous methods [42]. The resulting MS/MS data were processed using the MaxQuant search engine (v.1.5.2.8). Tandem mass spectra were searched against the SwissProt Mouse database concatenated with a reverse decoy database. Trypsin/P was specified as a cleavage enzyme, allowing up to four missing cleavages. The mass tolerance for precursor ions was set as 20 ppm in the first search and 5 ppm in the main search, and the mass tolerance for fragment ions was set as 0.02 Da. Carbamidomethyl on Cys was specified as a fixed modification, and 2-hydroxyisobutyrylation modification and oxidation on Met were specified as variable modifications. FDR was adjusted to <1%, and the minimum score for modified peptides was set at >40.

### Plasma metabolic profiling

Plasma metabolic profiling based on NMR measurements was using the same methodology used in the previous paper [26]. The glucose content in Fig. 1G comes from the plasma metabolic profiling.

### Quantitative analysis of energy metabolites in mouse heart tissue by LC-MS/MS

Quantitative analysis of energy metabolites based on LC-MS/MS was using the same methodology used in the previous paper [26]. The content of other metabolites except for glucose in Figs. 1G and 2E comes from the quantitative analysis of energy metabolites in mouse heart tissue by LC-MS/MS.

### Immunoprecipitation

Fresh cardiac tissues or cells were washed twice with 1×PBS and lysed with RIPA buffer (Beyotime Biotechnology, Shanghai, China) containing a protease inhibitor mixture (Beyotime Biotechnology). The extracts were centrifuged at 12,000×*g* for 15 min at 4 °C. Equal amounts of extracts were incubated with anti-2-hydroxyisobutyrylation-lysine agarose beads (Hangzhou Jingjie Biotechnology Co., Ltd, Hangzhou, China) or antibodies against Flag (Sigma), DLD (Abcam, Cambridgeshire, England), and P300 (Abcam) at 4 °C on a rotation wheel overnight. The lysates were then incubated with protein-G/A agarose beads for 2 h. The resulting protein complexes were washed with lysis buffer, separated by sodium dodecyl sulfate–polyacrylamide gel electrophoresis (SDS-PAGE), and immunoblotted with specific primary antibodies.

### Western blotting

For western blot analysis, protein homogenates from heart tissues or cells were separated by SDS-PAGE and transferred to a PVDF membrane. After blocking with 5% non-fat milk for 2 h, the membrane was incubated with antibodies against DLD (Abcam), P300 (Abcam), 2-hydroxyisobutyryl-lysine (Hangzhou Jingjie Biotechnology Co., Ltd), Flag (Sigma), HA (Sigma), and β-actin (Abcam). Subsequently, the membrane was incubated with HRP-conjugated secondary antibodies (Beyotime Biotechnology) and developed using a chemiluminescent substrate (Beyotime Biotechnology).

### Animal transfer of the AAV9

The vector (AAV9-GV469) and p300 overexpression (AAV9-GV469-p300) viruses were constructed by Shanghai Jikai Gene Technology Co., Ltd (Shanghai, China). The mice received an intra-myocardial injection with 8 × 10^10^ viral genomes of AAV9-GV469 and AAV9-GV469-p300 viruses using a 30-gauge needle.

### Multiple sequence alignment

The DLD protein sequences of different species were searched on the NCBI website (https://www.ncbi.nlm.nih.gov/), and the FASTA file of the protein sequence was downloaded. Multiple sequence alignments were performed using Clustalw in the MEGA software. After the comparison was completed, the comparison result file in FASTA format was exported for subsequent analysis. The sequence alignment results were displayed using GeneDoc software.

### Site-directed mutagenesis and purification of DLD

The pET28a-DLD vector was structured for expression, and this primer was used to lone DLD gene: DLD-sense: 5′-CGCGGATCCATGCAGAGCTGGAGTCGT-3′, DLD-antisense: 3′-CCCAAGCTTTTAAAAGTTGATTGGTTTCC-5′, and BamHI and XhoI were selected as restriction enzyme cutting sites to insert DLD into pET28a and the constructed vectors were transformed into E. coli BL21 (DE3) for the protein expression. Next, the mutated sites of DLD gene were introduced into the pET28a-DLD by PCR reaction, and the primers were given below: K277R-sense: 5′-AATACACGAGTTACTGGTGCCACC-3′, K277R-antisense: 3′-GTAACTCGTGTATTCAGTTTAAACT-5′; K277T-sense: 5′-ATACAACAGTTACTGGTGCCACCA-3′, K277T-anti-sense: 3′-GTAACTGTTGTATTCAGTTTAAACTT-5′; K277A-sense: 5′-ATACAGCAGTTACTGG TGCCACCAA-3′, K277A-antisense: 3′-GTAACTGCTGTATTCAGTTTAAACTT-5′; K288R-sense: 5′-GATGGACGAATTGATGTGTCTGTCG-3′, K288R-antisense: 3′-TCAATTCGTCCATCTGACTTCTTGGT-5′; K288T-sense: 5′-GATGGAACAATTGATGTGTCTGTCG-3′, K288T-antisense: 3′-TCAA TTGTTCCATCTGACTTCTTGGT-5′; K288A-sense: 5′-GATGGAGCAATTGATGTGTCTGTCG-3′, K288A-antisense: 3′-TCAATTGCTCCATCTGACTTCTTGGT-5′; K440R-sense: 5′-TGGTGCGGAT TCTTGGACATAAGTC-3′, K440R-antisense: 3′-AGAATCCGCACCATGCCATCTGTGT-5′, K440T-sense: 5′-TGGTGACGATTCTTGGACATAAGTC-3′, K440T-antisense: 3′-AGAATCGTCACCATGCCATCTGTGT-5′; K440A-sense: 5′-TGGTGGCGATTCTTGGACATAAGTC-3′, K440A-antisense: 3′-AGAATCGCCACCATGCCATCTGTGT-5′. E. coli BL21/pET28a-DLD was incubated in 5 mL LB medium containing ampicillin (50 μg/mL) overnight at 37 °C in shaking flasks, and then the cultures were transformed into 500 mL of fresh LB medium with ampicillin (50 μg/mL) at 37 °C in shaking flasks to an optical density at 600 nm of 0.6–0.8. Cells were induced with IPTG (0.15 mM) at 37 °C for 4 h. The proteins were harvested from cultured cells using the lysis buffer (20 mM Tris-HCl, pH 8.0, 10 mM MgCl_2_, 1 mg/mL lysozyme, and 50 U/mL nucleases). The proteins were mixed with HisPur Ni-NTA resin and washed with wash buffer (20 mM Na_3_PO_4_, 300 mM NaCl, 25 mM imidazole, pH 7.4), then eluted with the elution buffer (20 mM Na_3_PO_4_, 300 mM NaCl, 250 mM imidazole, pH 7.4). The elution was collected and concentrated using an Amicon Ultra-0.5 Centrifugal Filter Device in a storage buffer (100 mM HEPES, 10 mM MgCl2, 7.7 mM KCl, pH 7.0).

### Surface plasmon resonance analysis

In this study, a Biacore T200 optical biosensor (GE Healthcare, USA) was used for the surface plasmon resonance (SPR) experiment. Series S CM5 chips (GE Healthcare, USA) were selected as coupling chips. Ethanolamine HCl, EDC, NHS, caps, and sampling vials were all purchased from Biacore (GE Healthcare, USA). The running buffer was PBS buffer contains 5% DMSO. The recombinant DLD wild-type and mutant proteins (about 50 mg/mL) were coupled to a CM5 chip with approximately 7000 RU coupling amount for each channel. Then the diluted Rg3 (0–300 μM) was flowed through the chips at 30 μL/min. The solvent correction was performed at the same time, and the data were collected and analyzed in the Biacore T200 evaluation software.

### Adult cardiomyocyte isolation

Ventricular myocyte was isolated from the mouse hearts using a previously described method [43]. Mice were injected with heparin (50 units) and sacrificed by cervical dislocation. The heart was quickly removed from the chest, and the aorta was retrogradely perfused at 37 °C for 3 min with calcium-free Tyrode buffer (137 mM NaCl, 5.4 mM KCl, 1 mM MgCl_2_, 10 mM glucose, 10 mM HEPES, 10 mM 2,3-butanedione monoxime (Sigma), and 5 mM taurine (Sigma) at pH 7.4) gassed with 95% O_2_ and 5% CO_2_. Enzymatic digestion was initiated by adding collagenase type B (0.35 U/ml; Roche, Basel, Switzerland) and hyaluronidase (0.1 mg/ml; Sigma) to the perfusion solution. When the heart became swollen after 10 min of digestion, the left ventricle was quickly removed, cut into several chunks, and further digested in the same enzyme solution on a shaker (60–70 rpm) for 10 min at 37 °C. The supernatant containing the dispersed myocyte was filtered through a cell strainer (100 µm diameter; BD Falcon, New York, USA) and gently centrifuged at 500 rpm for 1 min. Extracellular calcium was incrementally added back to a final concentration of 1.25 mM over 30 min to avoid the calcium paradox.

### PDH activity assay

PDH activity was determined to use an assay based on the reduction of NAD^+^ to NADH, as described by the manufacturer.

### Acetyl-CoA content, pyruvate content, and lactate content assay

The manufacturer determined acetyl-CoA, pyruvate, and lactate contents (BC0980/BC2205/BC2235, Beijing Soleibao Technology Co., Ltd., Beijing, China).

### Simulation of binding of Rg3 and P300

The binding modes of Rg3 and p300 were simulated using the molecular docking tool AutoDockTools and the molecular docking software AutoDock4. The PDB files of P300 were obtained from the PDB database. Small molecule information was searched and downloaded from the PubChem database (https://pubch-em.ncbi.nlm.nih.gov/). The AutoDock4 program was used for docking, and PyMOL was used to view molecular docking results.

### Histological and immunofluorescent assessments

Heart samples were excised, washed, and fixed with 4% phosphate-buffered formalin for 48 h. Tissues embedded in wax were cut into 5 μm slices. The sections were stained with hematoxylin and eosin (HE) and wheat germ agglutinin (WGA) for pathological morphological analysis. The fluorescence images were captured using an inverted fluorescence microscope (ECLIPSE, Nikon, Tokyo, Japan).

### Statistical analysis

The assays were performed three times. The experimental data were expressed as means ± SD. Statistical analysis was performed by one-way ANOVA or Kruskal–Wallis test of repeated experiments followed by SPSS 22.0 statistical software. A *p*-value < 0.05 was considered statistically significant.

## Supplementary information

Supplemental Material (WB)

Reproducibility checklist

## Data Availability

All data generated or analyzed during this study are included in this published article [and its supplementary information files].
