# Supplementary material for: Rg3 regulates myocardial pyruvate metabolism via P300-mediated dihydrolipoamide dehydrogenase 2-hydroxyisobutyrylation in TAC-induced cardiac hypertrophy
Source: Cell Death Dis. 2022 Dec 26;13(12):1073. doi: 10.1038/s41419-022-05516-y (PMC9792576; doi:10.1038/s41419-022-05516-y)
Supplement: Supplementary file 1 — Supplemental Material (WB) [file 41419_2022_5516_MOESM1_ESM.docx]

**Original full length western blots**

**Fig 2F**

**
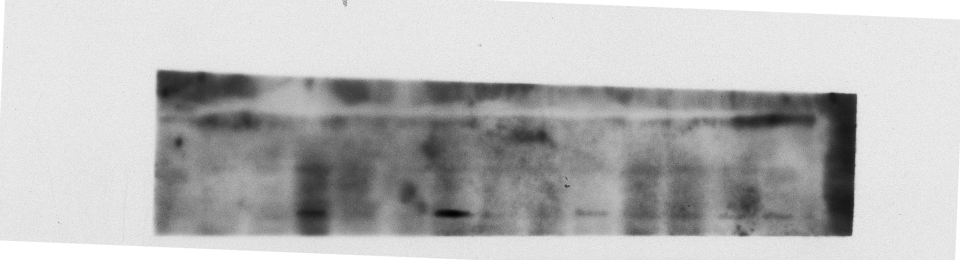
**

**
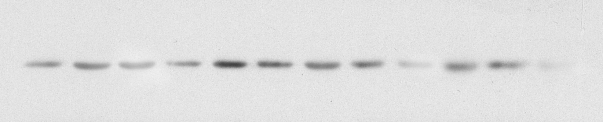
**

**
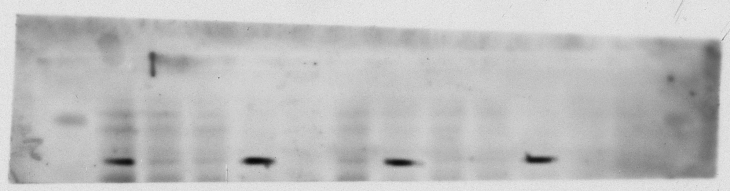
**

**Fig 4A**


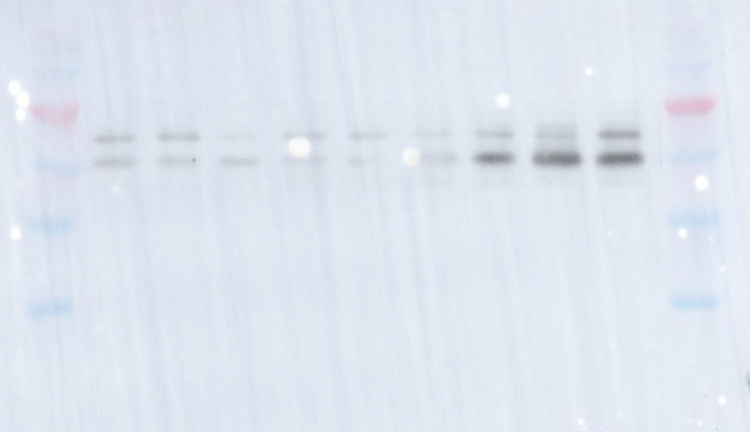


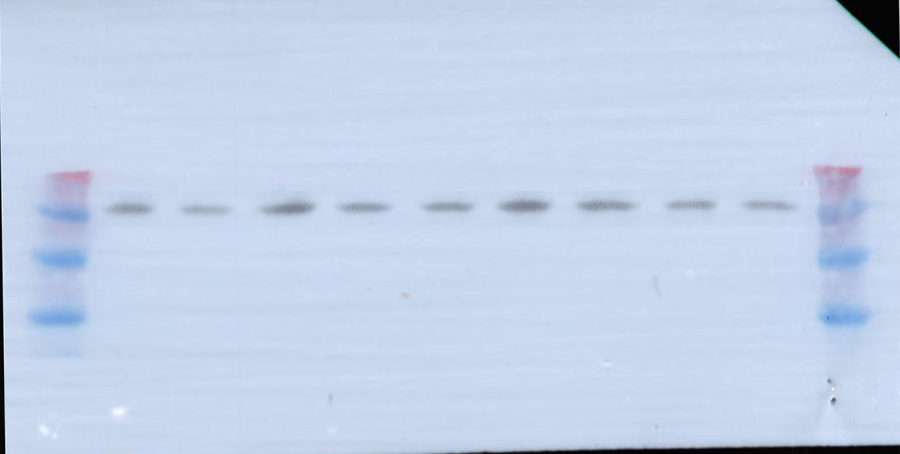


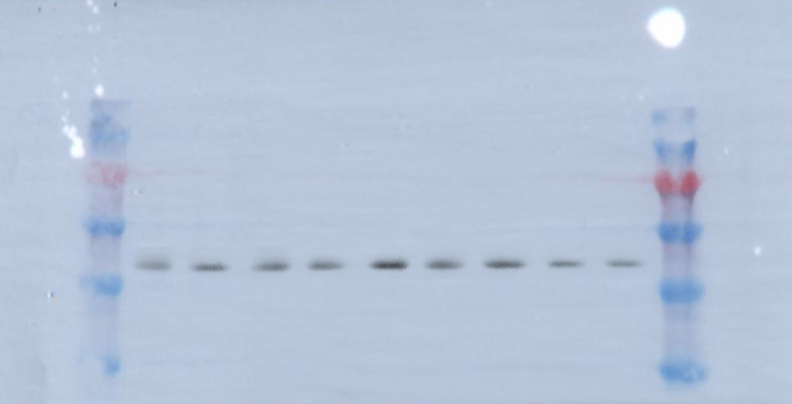


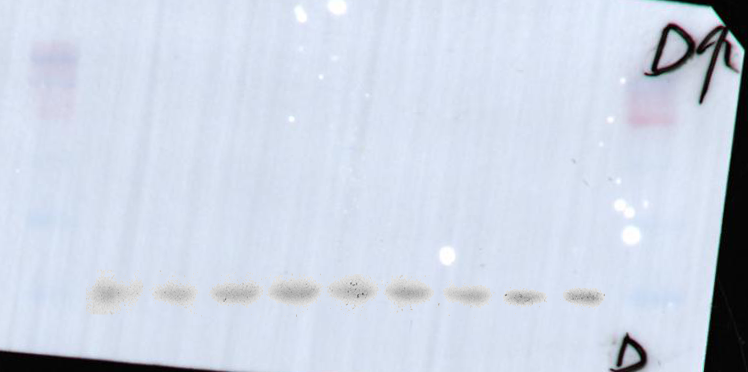


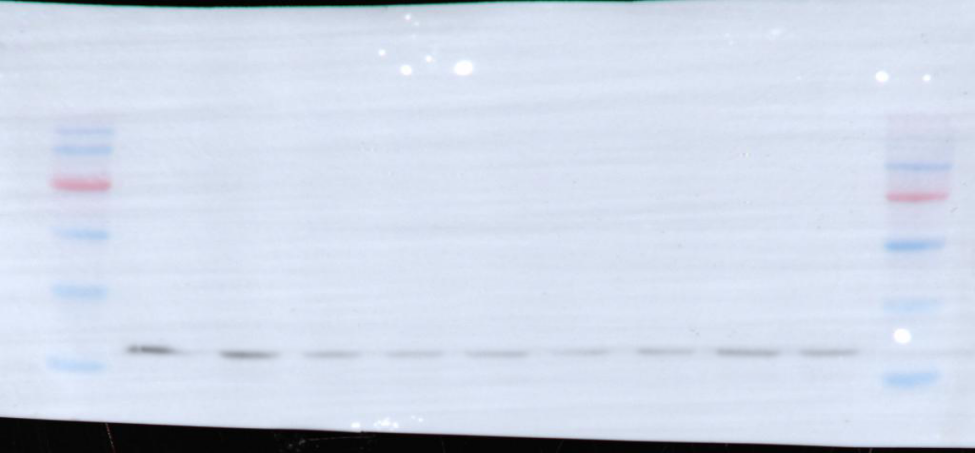


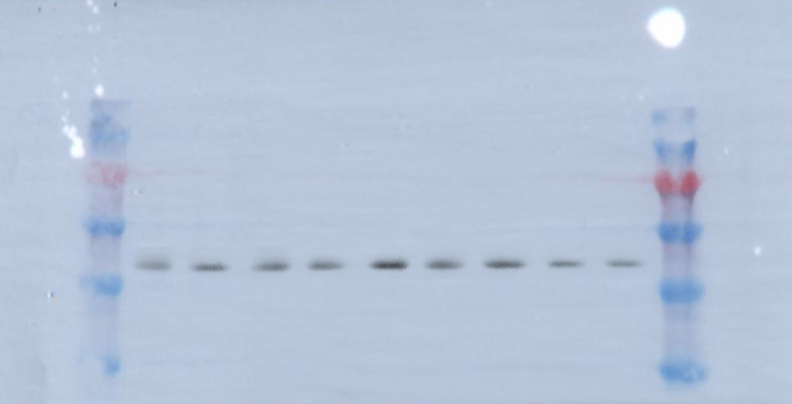


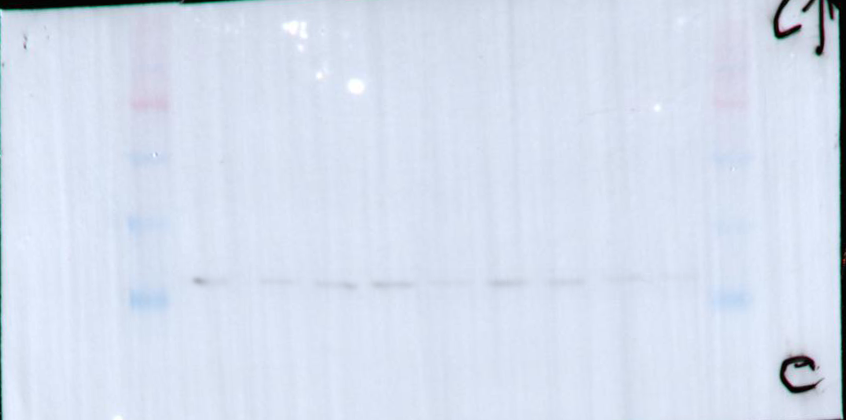


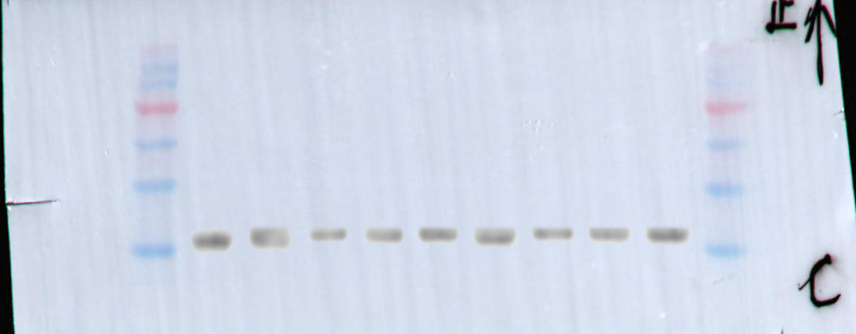


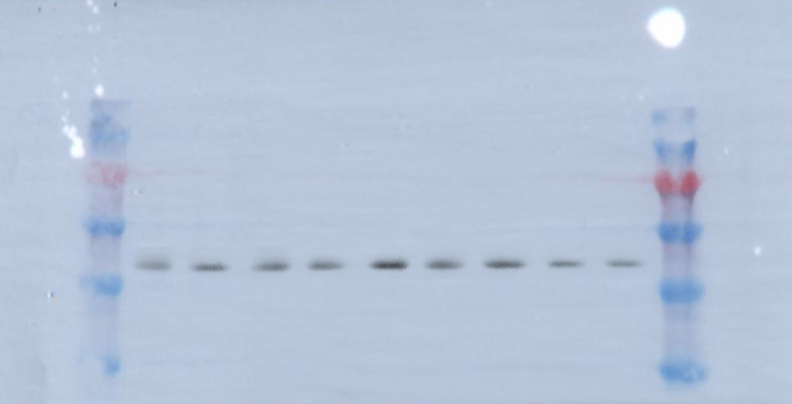


**Fig 4B**

**
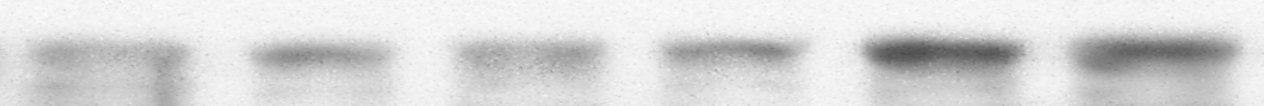
**

**
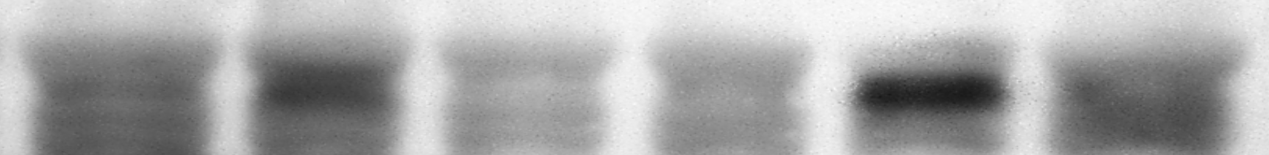
**

**
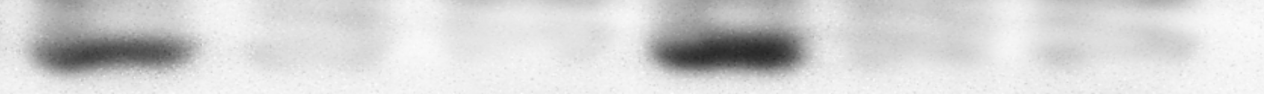
**

**Fig 4D**

**
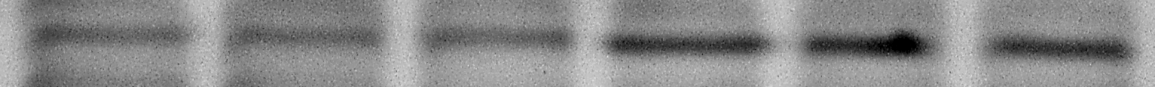
**

**
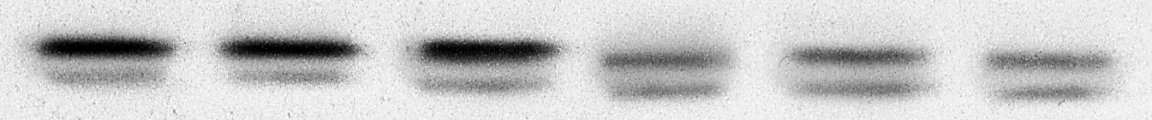
**

**
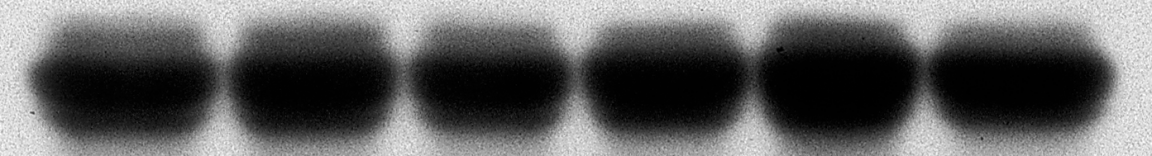
**

**Fig 4F**

**
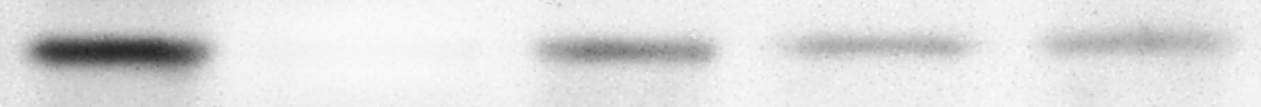
**

**
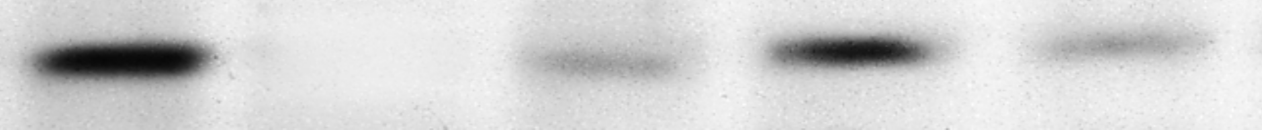
**

**
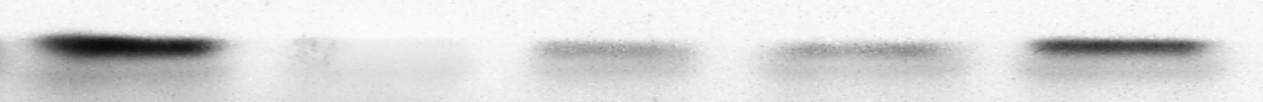
**

**
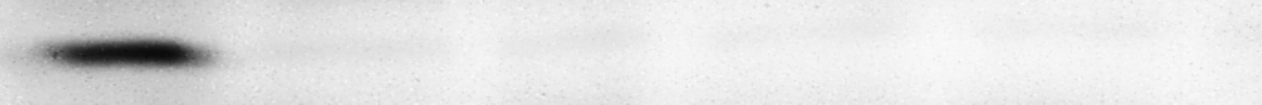
**

**Fig 4G**

**
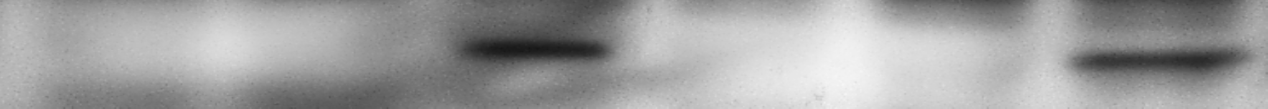
**

**
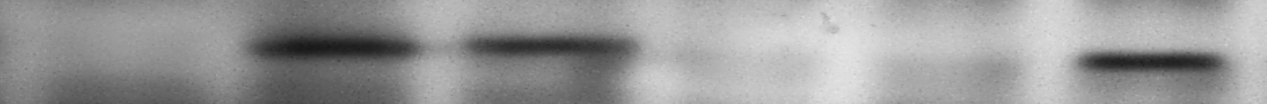
**

**
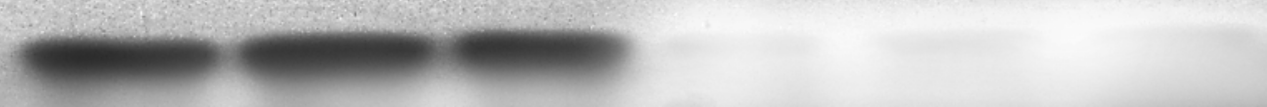
**

**Fig 5A**

**
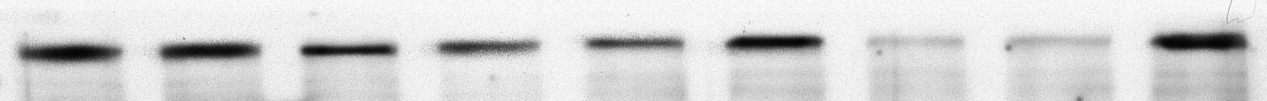
**

**
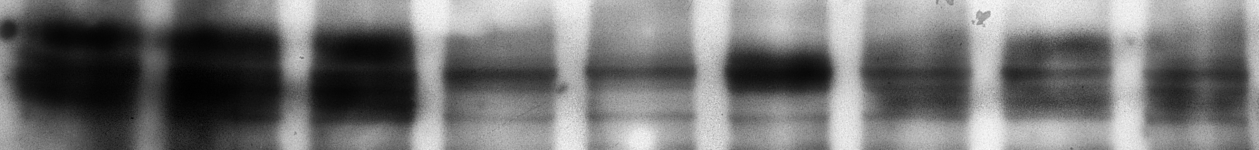
**

**
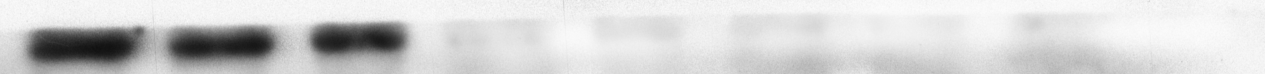
**

**Fig 5B**

**
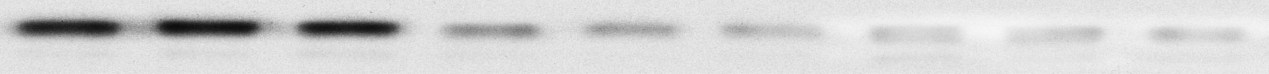
**

**
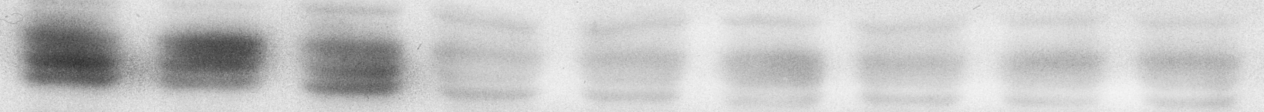
**

**
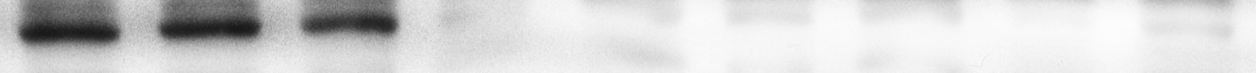
**

**Fig 5C**

**
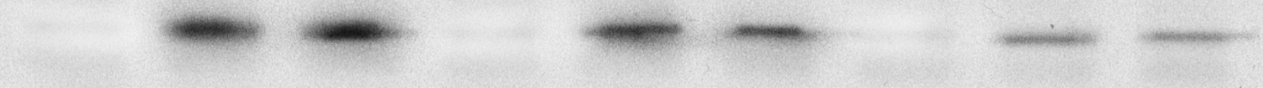
**

**
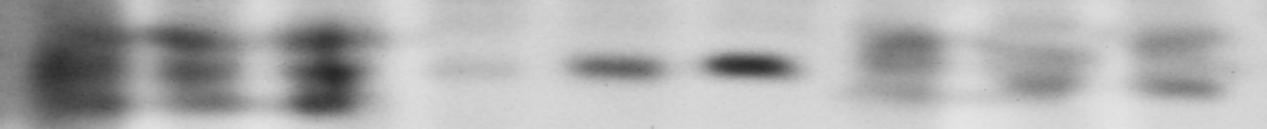
**

**
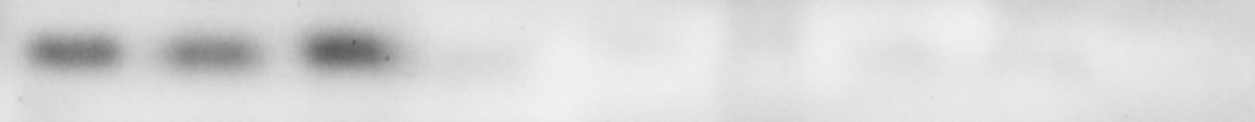
**

**Fig 5D**

**
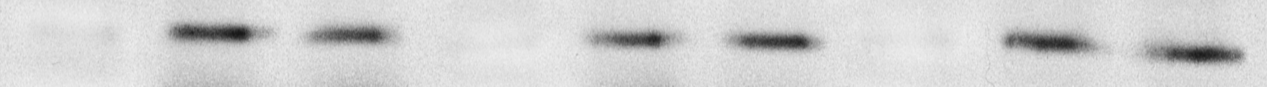
**

**
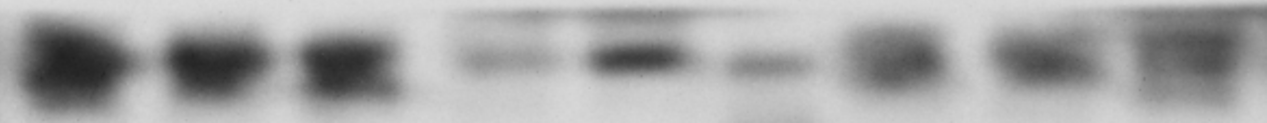
**

**
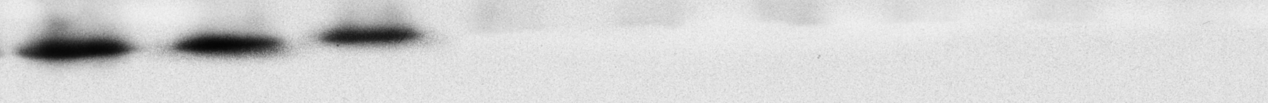
**

**Fig 6A**

**
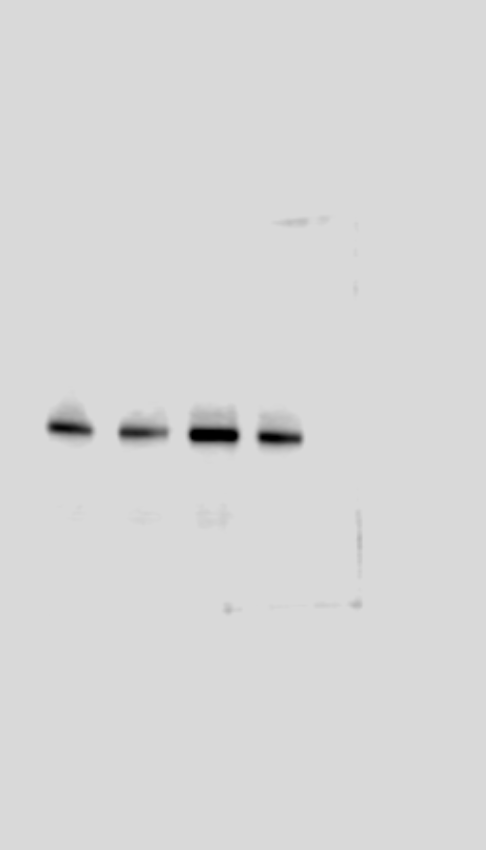
**

**
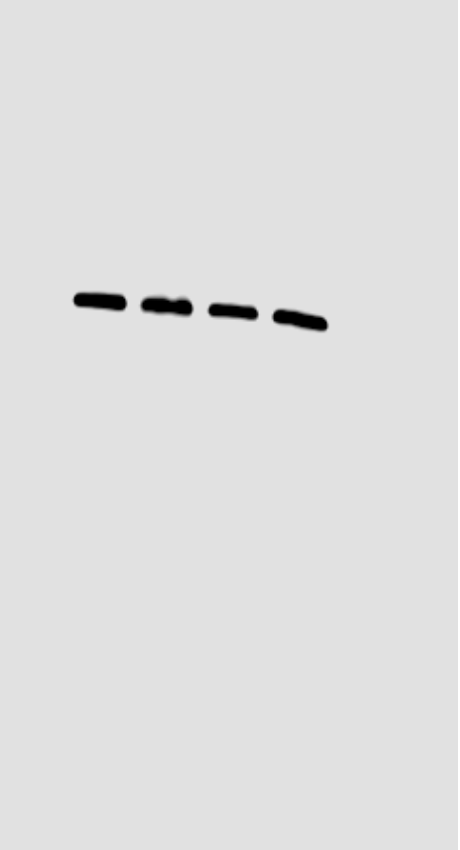
**

**Fig 6D**

**
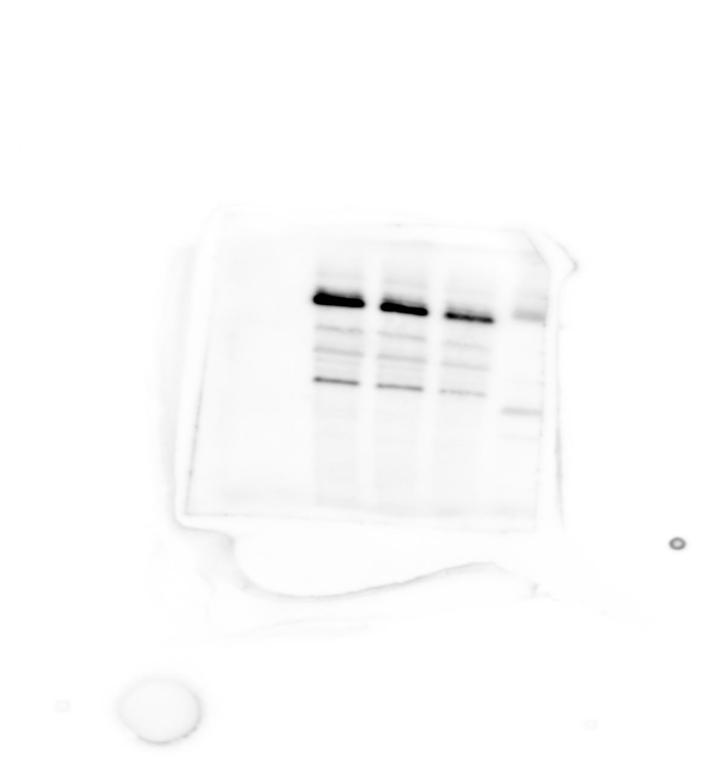
**

**
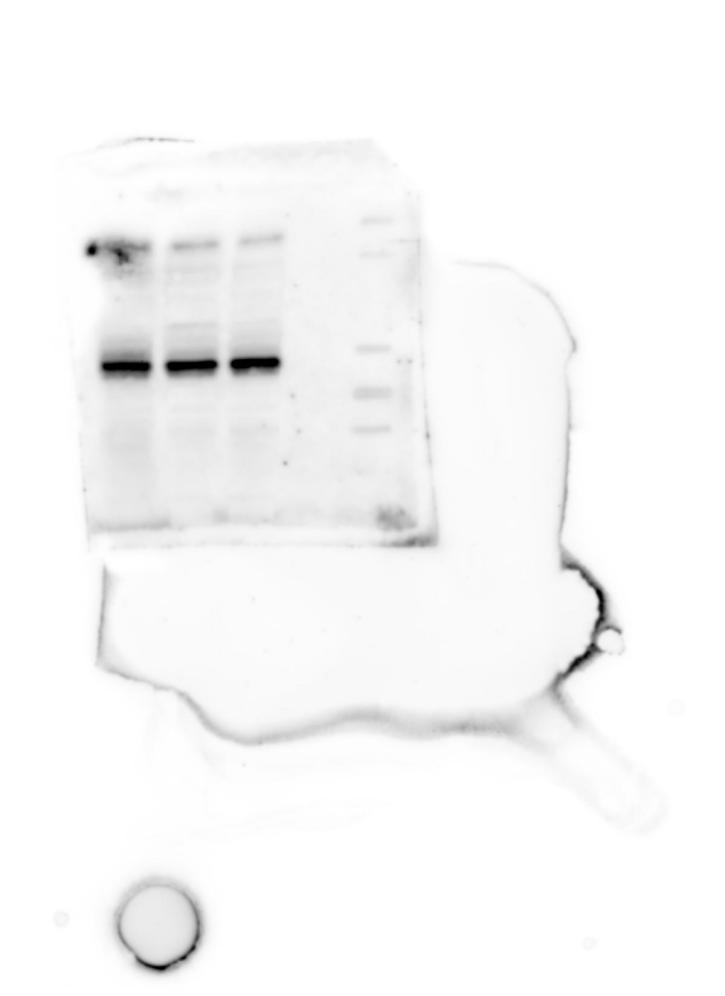
**

**Fig 6E**

**
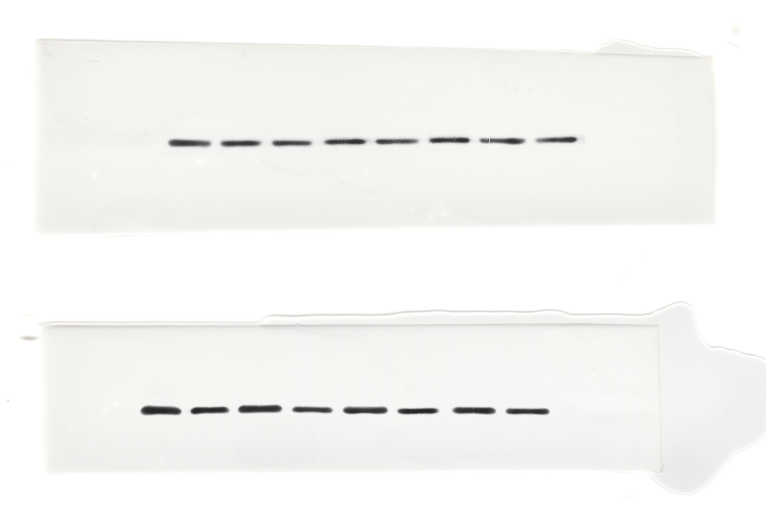

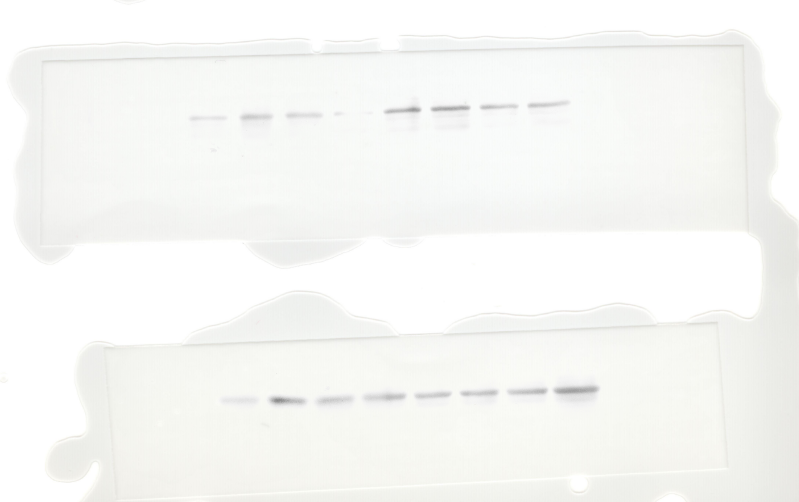

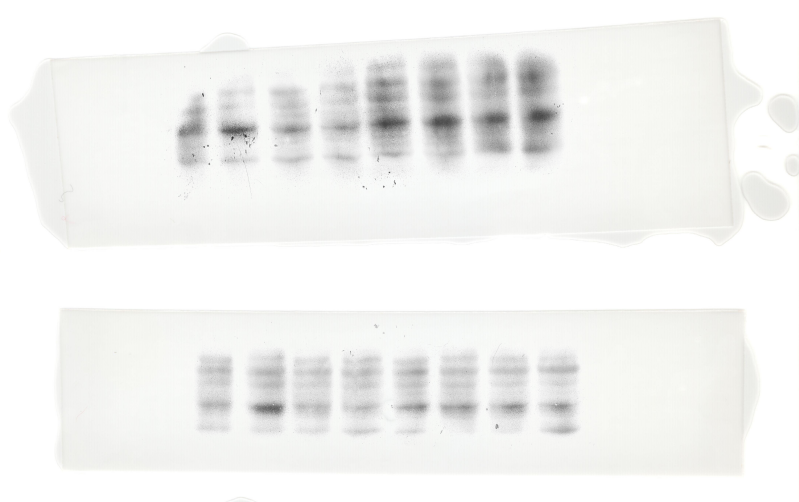
**
